# Quantitative stiffness assessment of cardiac grafts using ultrasound in a porcine model: A tissue biomarker for heart transplantation

**DOI:** 10.1016/j.ebiom.2022.104201

**Published:** 2022-08-03

**Authors:** Olivier Pedreira, Clement Papadacci, Lionel Augeul, Joseph Loufouat, Mégane Lo-Grasso, Mickael Tanter, René Ferrera, Mathieu Pernot

**Affiliations:** a Physics for Medicine, ESPCI, INSERM U1273, CNRS UMR 8063, PSL University, Paris, France; b CarMeN, 27102INSERM U1060, INRA U1397, INSA de Lyon, Université Claude Bernard Lyon 1, Université de Lyon, Villeurbanne, France

**Keywords:** Ultrasound, Shear wave elastography, Cardiac transplantation, Medical imaging

## Abstract

**Background:**

Heart transplantation is the definitive treatment for many cardiovascular diseases. However, no ideal approach is established to evaluate heart grafts and it mostly relies on qualitative interpretation of surgeon based on the organ aspect including anatomy, color and manual palpation. In this study we propose to assess quantitatively the Shear Wave Velocity (SWV) using ultrasound as a biomarker of cardiac viability on a porcine model.

**Methods:**

The SWV was assessed quantitatively using a clinical ultrasound elastography device (Aixplorer, Supersonics Imagine, France) linked to a robotic motorized arm (UR3, Universal Robots, Denmark) and the elastic anisotropy was obtained using a custom ultrasound research system.

SWV was evaluated as function of time in two porcine heart model during 20h at controlled temperature (4°C). One control group (*N* = 8) with the heart removed and arrested by cold cardioplegia and immerged in a preservation solution. One ischemic group (*N* = 6) with the organ harvested after 30 min of *in situ* warm ischemia, to mimic a donation after cardiac death. Hearts graft were revived at two preservation times, at 4 h (*N* = 11) and 20 h (*N* = 10) and the parameters of the cardiac function evaluated.

**Findings:**

On control hearts, SWV remained unchanged during the 4h of preservation. SWV increased significantly between 4 and 20h. For the ischemic group, SWV was found higher after 4h (3.04 +/- 0.69 vs 1.69+/-0.19 m/s, *p* = 0.007) and 20h (4.77+/-1.22 m/s vs 3.40+/-0.75 m/s, *p* = 0.034) of preservation with significant differences. A good correlation between SWV and cardiac function index was found (r^2^=0.88) and manual palpation score (r^2^=0.81).

**Interpretation:**

Myocardial stiffness increase was quantified as a function of preservation time and harvesting conditions. The correlation between SWV and cardiac function index suggests that SWV could be used as a marker of graft viability. This technique may be transposed to clinical transplantation for assessing the graft viability during transplantation process.

**Funding:**

FRM PME20170637799, Agence Biomédecine AOR Greffe 2017, ANR-18-CE18-0015.

**Research in context**

**Evidence before this study**

Non-invasive assessment of explanted cardiac grafts remains challenging as there is no ideal approach established. Ex situ assessment mostly relies on qualitative interpretation of surgeon based on the organ aspect including anatomy, color and manual palpation. Quantification of myocardial properties could be crucial in order to optimize preservation strategies, harvesting technique and finally to expand the number of grafts available for cardiac transplantation.

**Added value of this study**

We demonstrated the capacity of ultrasound elastography to monitor grafts stiffness after heart harvesting, during the hypothermic preservation duration, and after warm ischemia. We show that SWV is a reliable tissue marker for monitoring the organ preservation as a function of time.

**Implications of all the available evidence**

This could become a powerful tool to optimize preservation strategies and also for graft viability estimation, especially those from DCD. Possibility of real time non-invasive measurements associated with easy implementation, strongly suggests that this technique may be considered for a future using during the transplantation process. SWE has the potential to provide a tissue biomarker of the heart viability and could offer a new accessible, portable and predictive tool before heart transplant.

Alt-text: Unlabelled box

## Introduction

Heart transplantation remains the definitive therapy for end-stage heart failure patients but the number of cardiac grafts available for transplantation is still largely insufficient.^1^^,^^2^ Various strategies have been proposed to expand the donor pool^3^^,^^4^ but many potential donors do not meet the criteria for transplantation. Increasing organ availability with donation after circulatory death (DCD) could be a promising option to overcome the shortage of cardiac grafts.^5^, ^6^, ^7^ However, potential organ damages induced by ischemia have raised concerns for the graft. Another possibility would be to extend the selection criteria, and many studies have focused on preservation strategies to extend the preservation period of 4-6h usually allowed for the heart.^8^ Nevertheless, in both cases, the assessment of the graft quality becomes critical because of the potential damages induced by the preservation and procurement conditions.

Non-invasive assessment of explanted cardiac grafts remains challenging as there is no ideal approach established. Ex situ assessment mostly relies on qualitative interpretation of surgeon based on the organ aspect including anatomy, color and manual palpation.^8^ Machine perfusion systems also offer the possibility to perform a global assessment of physiological (heart rate, electrocardiogram) and hemodynamic parameters (coronary flow rate, pressure).^9^, ^10^, ^11^ Analysis of biomarkers such as lactate has also been investigated but evidence for its predictive value is lacking, and its sensitivity may be sub-optimal.^12^, ^13^, ^14^ Non-invasive assessment using imaging methods such as MRI, CT and ultrasound (US) imaging have also been proposed to evaluate the tissue integrity at the local level. However, MRI and CT require long acquisition time and complex logistics which are not adapted to organ transplantation time race whereas conventional B-mode ultrasound imaging does not provide quantitative biomarkers of the tissues.

Ultrasound Shear Wave Elastography (SWE) has emerged during the last decade as a quantitative and non-invasive imaging modality to evaluate the viscoelastic properties of soft tissues.^15^, ^16^, ^17^ Stiffness is an important biomechanical property of the myocardium. Myocardial stiffness reflects alterations of cellular and extracellular components at the macro and microscopic scales. Several approaches based on acoustic radiation force (ARF) have been proposed to assess myocardial stiffness. ARFI relies on the assessment of tissue relaxation after applying an acoustic radiation force at one location,^18^ but this method does not provide an absolute estimate of myocardial stiffness. In contrast, shear wave elastography has been proposed for quantitative stiffness assessment by the measurement of the velocity of shear waves generated by the acoustic radiation force.^19^, ^20^, ^21^ Ultrafast imaging (>1000 images/) allowed imaging directly the propagation of the shear waves whereas other techniques such as pSWE relies on focused pulse echo estimations repeated several times.^22^ Because the velocity of shear waves is directly linked to tissue stiffness (shear modulus), SWE can provide quantitative maps of stiffness in real time. SWE has been successfully implemented for various applications such as the diagnostic of breast cancer, the non-invasive assessment of liver fibrosis and the characterization of thyroid.^23^^,^^24^ SWE has been extensively investigated in cardiac studies on animal models and more recently on human patients.^25^, ^26^, ^27^, ^28^, ^29^ Early alterations of myocardial stiffness have been shown during acute ischemia.^30^^,^^31^ Myocardial stiffness was also found to increase significantly in patients with myocardial alterations such as hypertrophic cardiomyopathy or amyloidosis patients. SWE was also recently proposed for the follow up of heart post-transplantation recipients.^32^

In this study, we propose to use SWE as a tool to assess cardiac grafts viability between heart harvesting and transplantation time. We developed a device to image an entire isolated porcine heart in different views to evaluate myocardial stiffness at several anatomical locations. A second ultrasonic system was also used to perform a multidirectional evaluation of the SWE.^33^ Thereby, new parameters can be evaluated such as the myocardium fibers parallel and perpendicular shear wave velocities or the fractional anisotropy.

## Methods

### Graft model, harvesting technique and preservation

Experiments were performed in explanted hearts of a porcine model. The animals used are healthy, young adults, non-genetically modified farm pigs of both genders, weighing 30-35 kg, supplied by breeders approved by the veterinary authorities.

To limit the stress of the animal and accustom it to human presence, daily care has been provided (caresses, games with the animal attendant). After housing and acclimatization period of 72h in an approved animal facility (water and food *at libitum*, enrichments and controlled environmental conditions), the animals were anesthetized as follows: induction of anesthesia was performed by an IM injection of a mixture of Ketamine (6.5 mg/kg) and Xylazine (1.5 mg/kg). After introduction of cathlon (24G 11/10) into a marginal vein of the ear, a bolus of propofol (1%, 200µL/kg) and Fentadon (0.035μg/kg) is injected by IV, in order to complete the anesthesia. The installation of a 7G tracheal probe, connected to an Alpha100 type respirator (Frequency: 15/min - Tidal volume: 6L / min - O_2_ / Air mixture: 50/50) ensures assisted ventilation and maintenance of the volatile anesthesia (with 2.5% Sevoflurane).

After stabilization of the hemodynamic parameters (cardiac and respiratory indices, evaluating the absence of pain and the depth of anesthesia), a left thoracotomy was performed and the hearts were randomly allocated to one of the 2 following groups, at the end of the surgical preparation to limit confounding factors related to the animal. In the control group (*N* = 8), the heart was arrested by cold (4°C) cardioplegia and rapidly immerged in a preservation solution. In the second group (*N* = 6) heart harvesting was preceded by 30 min of in situ warm ischemia, to imitate a Maastricht 3 protocol (where the grafts are removed, at the express condition that a cardiorespiratory downtime of approximately 30 min is observed). Due to the differences between the 2 types of surgical preparation, all participants were aware of the attribution of the groups.

After harvesting and cooling, each heart was suspended by the aorta and immerged on a preservation solution. This preservative technique was chosen due to simplicity and reproducibility without risk for the graft. The preservation solution used called LYPS was developed by Unité de recherche INSERM-UMR1060, Lyon, France. This preservation solution is mainly composed of Na^+^ (110 mmol/L), K^+^ (20 mmol/L), Ca^2+^ (1 mmol/L), Cl^–^ (150 mmol/L), Mg^2+^ (4 mmol/L) and metabolic agents. More details can be found in Michel et al.^34^

Each graft was preserved during 20h at controlled temperature and their stiffness was measured three times. At first Shear Wave Velocity (SWV) measurement, which is the velocity of the traveling shear wave, were performed right after graft immersion as a reference point of a fresh harvested organ. A second set of measurements was obtained after 4h of preservation which corresponds to the time limit for organ transplantation. A last set of measurements was finally performed 20h after the harvesting in order to evaluate long preservation time impact on graft stiffness.

Of all the experiments, approximately 10% of the animals were excluded for the following reasons: (i) surgical problem (for example, aorta too short or damaged at the time of sampling, injury to the pulmonary artery resulting in significant initial hemorrhage and death of the animal), (ii) problem related to a pathology of the animal (for example, aortic insufficiency, infections, sepsis).

The measurements and analyzes were not carried out on the excluded animals.

### Ethics

This study received the agreement by the French national ethics committee: APAFIS N°16758-201809171143620. All animals received humane care in compliance with the European Union Directive of 2010 (2010/63/EU), and the study was approved by the institutional and regional committees for animal care and followed the ARRIVE guidelines.

### Evaluation of cardiac function

At the end of the cold storage period, 13 control hearts and 8 ischemic hearts were prepared for the reperfusion step and assessment of cardiac function. 7 control and 4 ischemic hearts were preserved during 4h and 6 control and 4 ischemic hearts were preserved during 20h at 4°C. It should be noted that the heart revived were preserved and harvested in the same conditions than the hearts previously scanned. The grafts were next revived using a perfusion solution at 37.5°C, in practice, a latex balloon was introduced into the left ventricle and secured in the mitral annulus. The balloon was then connected to a pressure transducer and inflated with water to a diastolic left ventricular pressure of 5 mmHg. Hearts were submitted to aortic reperfusion with 600 ml of conserved heparinized whole blood at 38°C. The initial pH of the solution was 7.2-7.4 after equilibrium with a mixture of O2/CO2 (95%-5%) and 20 ml of Na2CO3 at 4.2%. Perfusion pressure was gradually increased from 2.9 KPa (30 cm H2O) at the beginning of reperfusion to 4.9 KPa (50 cm H2O) at 20 min to 30 min. Calcium was added after 20 min of reperfusion to obtain a final mean calcium level of 2.5 ± 0.19 mmol/l. At baseline and after 15, 30, 45 and 60 min of reperfusion, the functional state of the heart was evaluated by measuring Left Ventricular Developed Pressure (LVDP), heart rate (HR), RPP (= LVDP.HR), myocardial contractility and relaxation rate, and end diastolic pressure (EDP).

### Experimental set up for US imaging

**Figure 1:**
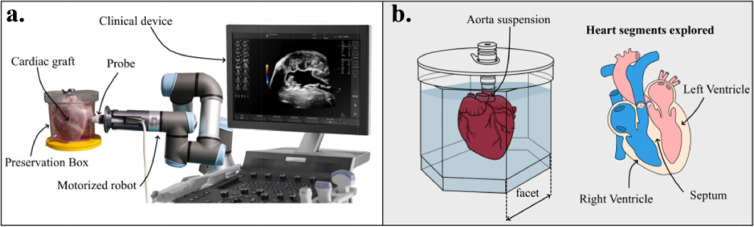
Experimental set-up. **a.** The heart graft was immerged on an ultrasound transparent box suspended by the aorta. The robot system was used to hold the imaging probe and enable scanning. Three heart structures were explored, the right ventricle, the septum and the left ventricle. **b.** Preservation box and a slice view of the heart with the target structures for SWE analysis.

To perform ultrasound evaluation of the cardiac graft a dedicated preservation box minimizing the absorption and the reflection of ultrasound energy was manufactured. This preservation container has a hexagonal shape. Each facet enables longitudinal and transversal scans of the heart allowing a full exploration. The whole box was manufactured by Goodfellow (Lille, France) using a TPX polymer, material used for his low acoustic attenuation and low acoustic impedance mismatch.^35^ The robot was used to hold the ultrasound probe and scan the entire organ. One face of the box was unavailable for the US exploration because during manufacturing process a purging system was installed on it. A thin layer of ultrasound gel was used between the box side and the probe. Two different probes and ultrasound systems were used. A clinical ultrasound system, Aixplorer scanner (Supersonics Imagine, France), gold standard for SWE, with a linear probe (SL10-2, Supersonics Imagine) coupled to a rotational robot (UR3, Universal Robots, Denmark) to perform conventional ultrasound images and SWE maps as represented on the Figure 1. A research system developed in house and described in Pedreira et al. was also used to assess the elastic anisotropy. The probe was maintained by a mechanical fixation arm. We defined ten regions of interest in the left ventricular, right ventricular and septal regions from apex to base as show on the right of the figure.

We first performed a scan of the whole heart, B-mode images from short axis and long axis views were acquired on each side of the preservation box. On each of the faces 91 slices were obtained along long axis view at steps of 1mm and 95 slices along the short axis with the same step. The acquisition is automatic and each face of the box can be entirely scanned in 4 min. Cardiac structures and segments were labelled by a trained cardiologist and 10 segments were defined for SWE assessment. The right ventricle (RV) was imaged using three short axis slices, at basal, middle and the apical levels, and one long axis slice. Left ventricle (LV) was also explored using short axis slices at basal, middle, and apical levels and one long axis slice. Finally, the septum was imaged in short axis and long axis views.

### Shear wave velocity assessment

SWE maps obtained with Aixplorer system and the linear probe relies on two steps: firstly, a shear wave is remotely generated in the myocardium using acoustic radiation force, and secondly, plane wave images are transmitted at ultrafast frame rate to track the propagation of the shear wave and quantify shear wave velocity (SWV). The relationship between the stiffness measured by the shear modulus and the shear wave velocity can be found bellow:

(1) $$\mu =\rho c_{T}^{2}$$

With µ the shear modulus expressed in Pa, ρ [kg/m^2^] the density of the medium and $c_{T}$ [m/s] the SWV. It should be noted that in the case of an incompressible isotropic material, the Young modulus is proportional to the shear modulus (E∼3 µ). SWV was expressed in meters per second (m/s) and mapped as a color-coded two-dimensional SWV image from red for stiffer tissues to blue for soft tissues. A region of interest (ROI) of about 1 cm^2^ was defined using B-mode images to compute the mean and the standard deviation of the SWV estimates of each segment. We evaluated the myocardial stiffness of the 10 individual segments and defined also a global stiffness as the mean of all the individual values.

Because the myocardium is composed of fibers, the myocardial elastic properties are expected to be anisotropic. In clinical implementation of shear wave elastography, the medium is assumed to be isotropic. Therefore, to assess shear wave anisotropy, we used a dedicated research system, which was developed in our lab to assess myocardial stiffness in transverse isotropic medium such as skeletal muscle or the myocardium. The approach relies on a dedicated transducer developed in a previous study^33^ to measure simultaneously the SWV in three directions and provide the parallel and transverse SWV. The approach was validated using numerical simulations and experiments and was easily translated to this study. Fractional Anisotropy (FA) and the fibers orientation were assessed on one graft of each model.

### Overview of experimental sequence

In this study we aimed at evaluating the stiffness of two heart models (normal and ischemic) and the evolution of myocardial stiffness as a function of time during preservation. Three measurement time points were defined, each heart was preserved during a total time of 20h and the initial stiffness was measured right after the collect of the graft. A second measurement was performed after 4h of preservation. Each time the stiffness was evaluated on each segment of the graft as previously described. A last measurement was performed at 20h of preservation.

### Statistical analysis

To estimates differences, between the two studied group, the statistical analysis was performed using GraphPad Prism software (version 9.2.0, GraphPad Software Inc. USA). To analyze the stiffness evolution as a function of time, we used an ordinary one-way ANOVA with a Šidák multiple comparison the assumption of normality was verified using a Chi-square normality test due to the small number of samples. The comparison of stiffness between the two groups we performed using a two-way ANOVA design with a Šidák multiple comparison assuming independence between tests. Significance was assumed for *p* < 0.05. We also investigated the correlations between markers of the heart function in post reanimation and SWV values. We used a Pearson correlation assuming that values are sampled from populations that follow a Gaussian distribution and choosing a two-tailed P value. The p-value style was chosen as follow * *p* < 0.05, ** *p* < 0.01, *** *p* < 0.001

The sample size of the experiments was decided after performing a priori power test. The analysis was performed using nQuery (Statsols, USA) a clinical trial design software. The power analysis was performed using a t-test after the first sample of each model to estimate properly the two groups mean value (1.5 m/s / 2.0 m/s) and the common standard deviation (0.2 m/s) was estimated from literature and previous studies. The sample size predicted for a 95% power was found of *n* = 6.

### Role of the funding source

The funders did not have any role in study design, data collection, data analyzes, interpretation, or writing of report.

## Results

### Whole heart ultrasonic scan

**Figure 2:**
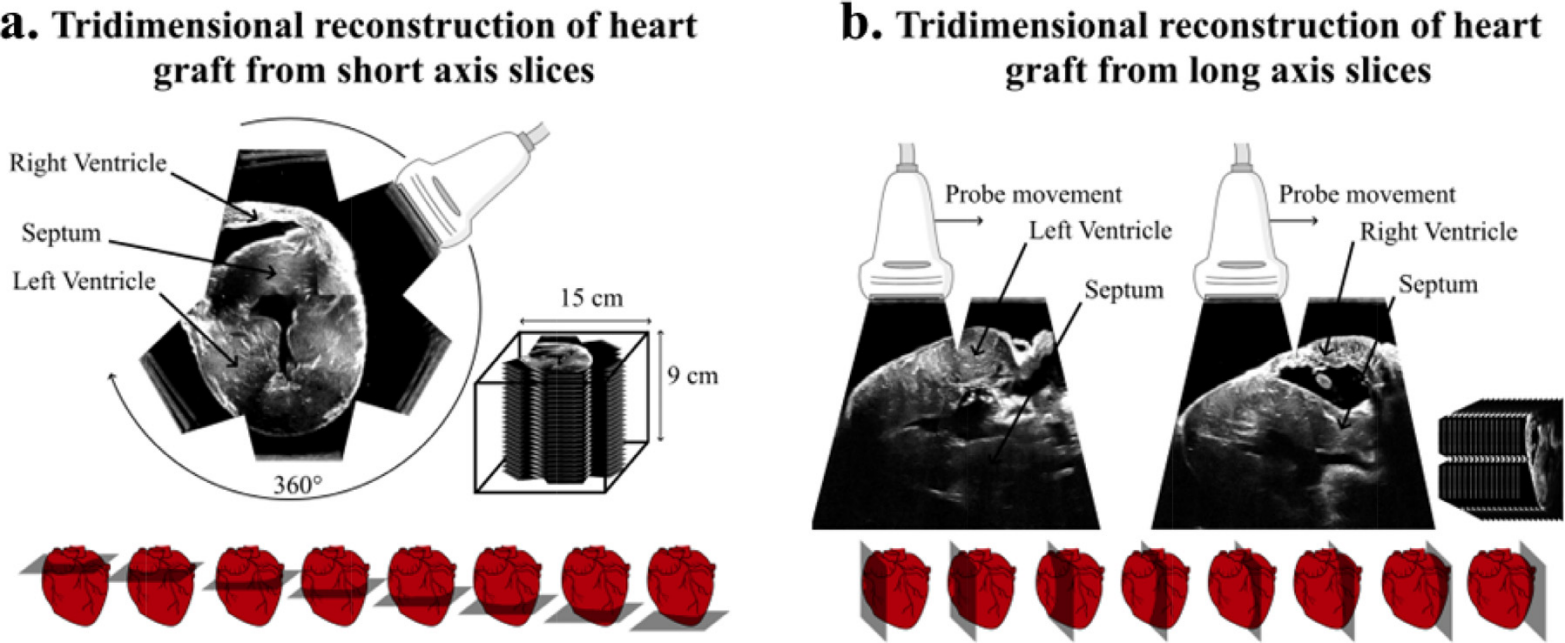
Images reconstruction of whole heart. **a.** Short axis view reconstruction from each face of the box along. **b.** Long axis view reconstruction of the right ventricle free wall side (left) and left ventricle free wall (right). One should note that heterogeneities of grayscale level is due to reconstruction artifacts and contact differences at the probe-box interface for each facet.

In order to identify the cardiac structures, we first acquired ultrasound slices of an immerged heart graft. Whole heart images were reconstructed by combining the different slices acquired. Figure 2 shows an example of reconstruction in short axis (A) and long axis (B) view of the septum, the left ventricle and the right ventricle. The size of the reconstructed volume was 150×150×90 mm.

### Stiffness evolution during hypothermic preservation of control hearts

**Figure 3:**
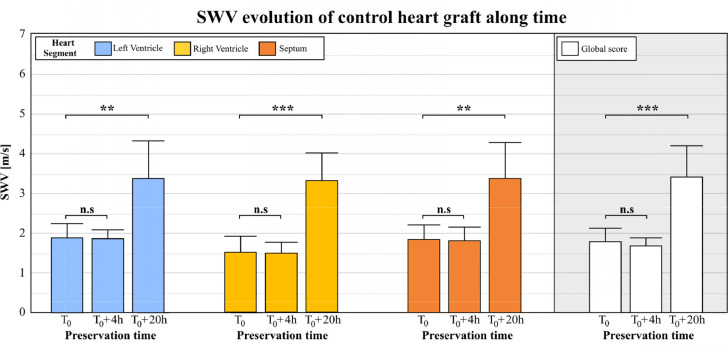
Evolution of SWV in different segments of eight control hearts as a function of time. From left to right, LV, RV and septal wall SWV boxplot at harvesting time T_0_ and after 4h and 20h of preservation. On the right, in white, the global SWV score of the organ.

First, we evaluated the evolution of myocardial stiffness in control hearts (*N*=8) as a function of preservation time. As shown on Figure 3, grafts SWV show an increase as a function of time and the same behavior was found for all the segments imaged, the LV, the RV and the septal walls. On the left of the figure, in blue, LV was evaluated at T_0_ (SWV = 1.87+/-0.35 m/s), after 4h (SWV = 1.86+/-0.23 m/s) and after 20h (SWV = 3.38+/-0.92 m/s). In yellow the same analysis was performed on the RV at T_0_ (1.52 +/- 0.37 m/s), after 4h (SWV = 1.50 +/- 0.24 m/s) and after 20h (SWV = 3.32 +/- 0.66 m/s). And finally, in orange, the septum was explored at T_0_ (SWV = 1.85+/-0.33 m/s), after 4h (SWV = 1.82+/-0.32 m/s) and after 20h (SWV = 3.38+/-0.88 m/s).

The fourth hour of preservation was compared with the SWE value at T_0_ defined as the harvesting time (one-way ANOVA with a Šidák multiple comparison). On each segment, no significant differences were observed on the LV (*p* = 0.392), the RV (*p* = 0.282) and septal wall (*p* = 0.334). The myocardial stiffness remained unchanged after 4h of preservation. Nevertheless, a strong stiffness increase was found between 0 and 20h and significant differences were found on the LV (*p* = 0.020), the RV (*p* = 0.002) and the septal wall (*p* = 0.018)

Measurements performed on each segment have been merged on the right of the figure to have a global evaluation of graft stiffness. A mean SWV value was computed for the three preservation times T_0_ (SWV = 1.78+/-0.3 m/s), 4h (1.69+/-0.19 m/s) and 20h (3.40+/- 0.75 m/s) representative of a global SWV value of the organ.

### Stiffness evolution of ischemic hearts

**Figure 4:**
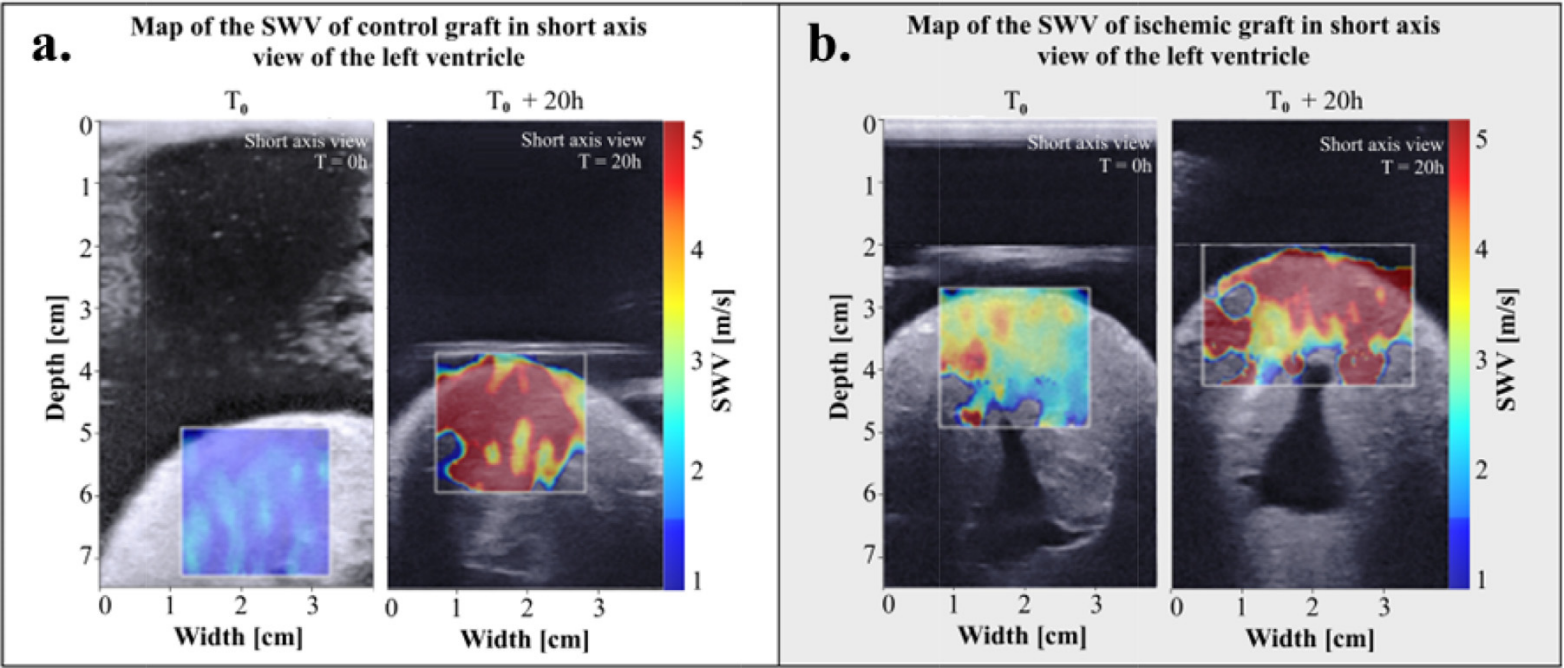
SWV colored map superimposed onto Bmode images of an ischemic heart graft right after harvesting and after 20h of preservation. **a.** The same control graft at the two preservation time and the same ischemic heart on **b**.

In the same way, we evaluated the stiffness evolution of the ischemic heart model as a function of time. Figure 4 shows an example of SWV maps of the left ventricular wall. Figure 4 (A) show the SWV map of one control graft at T_0_ and after 20h of preservation versus an ischemic graft on the Figure 4 (B). The SWV maps revealed a strong increase of myocardial stiffness due to preservation but also as function of harvesting model.

**Figure 5:**
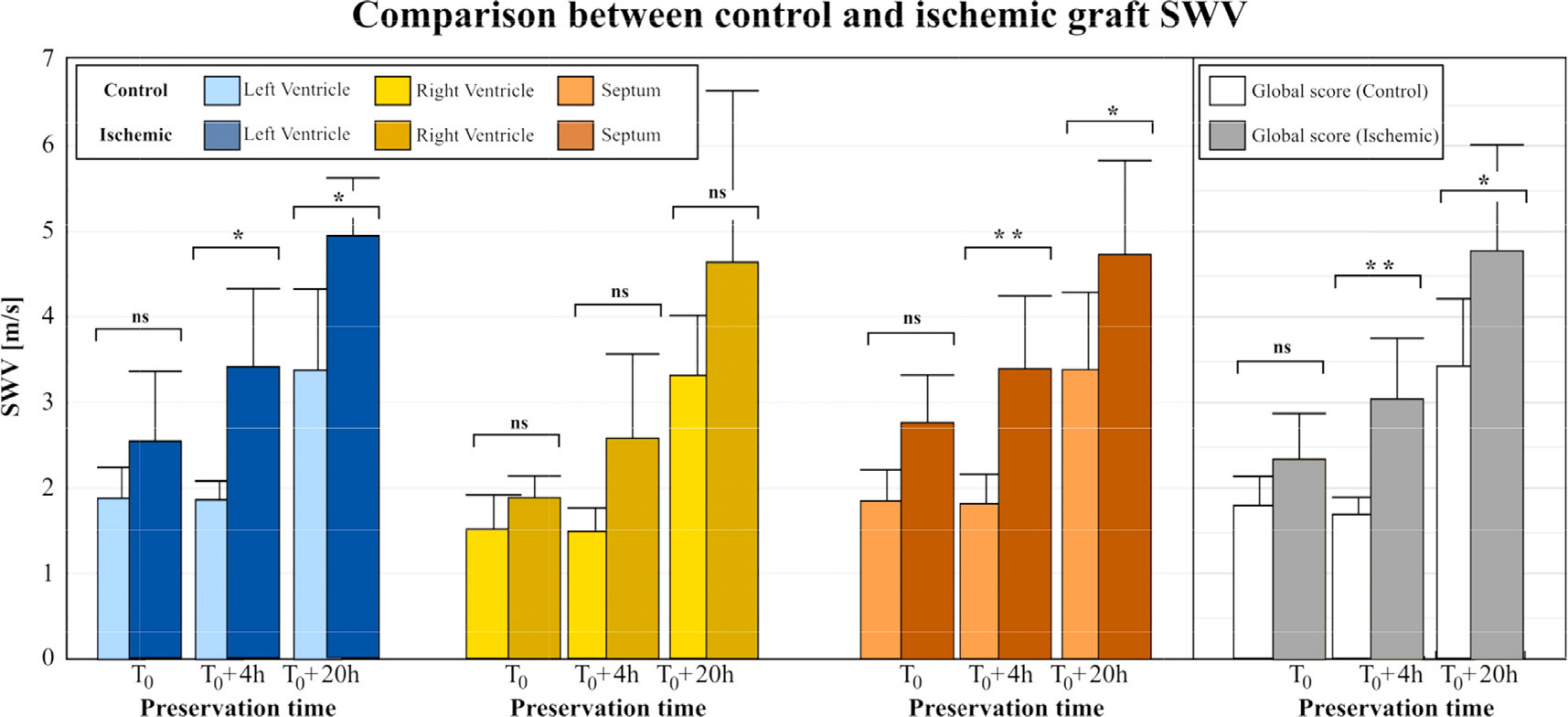
Boxplot comparison of the LV, the RV and the septal wall SWV of control grafts versus ischemic grafts at baseline, 4h and 20h of conservation. On the right the comparison of the global SWV score of the two models.

The analysis was performed on the 6 ischemic hearts (Figure 5), it also showed a myocardial stiffness increase between T_0_, 4h, and 20h of preservation. (two-way ANOVA design with a Šidák multiple comparison) We then compared the SWV between the two models and results are shown with a box plot.

In light blue, the SWV of the LV of control group was compared to ischemic group, in dark blue. Ischemic hearts were stiffer (2.54 +/- 0.79 m/s vs 1.87 +/- 0.35 m/s, *p*=0.211) at baseline, at 4h (3.41 +/- 0.89 m/s vs 1.86+/- 0.23 m/s, *p*=0.014) and at 20h (4.94 +/- 0.65 m/s vs 3.38+/- 0.66 m/s, *p* = 0.018).

The same observations can be done on the two other segments. The right ventricle, in yellow, show differences at T_0_ (1.89+/-0.24 m/s vs 1.52 +/- 0.37 m/s, *p* = 0.575) and after four hours of preservation (2.58 +/- 0.97 m/s vs 1.50 +/- 0.24 m/s, *p* = 0.227). No significant differences were found after 20h but we noticed a stiffer RV in the ischemic case (4.62 +/- 1.99 m/s vs 3.32 +/- 0.26 m/s, *p* = 0.078). Regarding the septal wall, represented in orange on the figure, a difference was found between the two models at each preservation time, after harvest (2.77 +/- 0.55 m/s vs 1.85 +/- 0.33 m/s, *p* = 0.351), at 4h (3.41 +/- 0.82 m/s vs 1.82 +/- 0.32 m/s, *p* = 0.004) and after 20h (4.75 +/- 1.03 vs 3.38 +/- 0.88 m/s, *p* = 0.049)

With a global evaluation of graft stiffness, mean SWV values were computed at T_0_ (2.35+/-0.53 m/s vs 1.78+/-0.3 m/s, *p* = 0.365), 4h (3.04 +/- 0.69 vs 1.69+/-0.19 m/s, *p*=0.007) and 20h (4.77+/-1.22 m/s vs 3.40+/-0.75 m/s, *p* = 0.034) with significant differences at 4h and 20h of preservation time compared to control.

### Evolution of the myocardial anisotropy

To analyze the stiffness alterations more finely, we performed experiments with a custom research system dedicated to the quantification of myocardial stiffness anisotropy. This new probe provides an estimation of the whole shear wave velocity using a multi-directional approach (see^33^). Using this system, two values of SWV were quantified: The parallel SWV (// SWV) along the fibers and the perpendicular SWV (⊥ SWV) across them.

**Figure 6:**
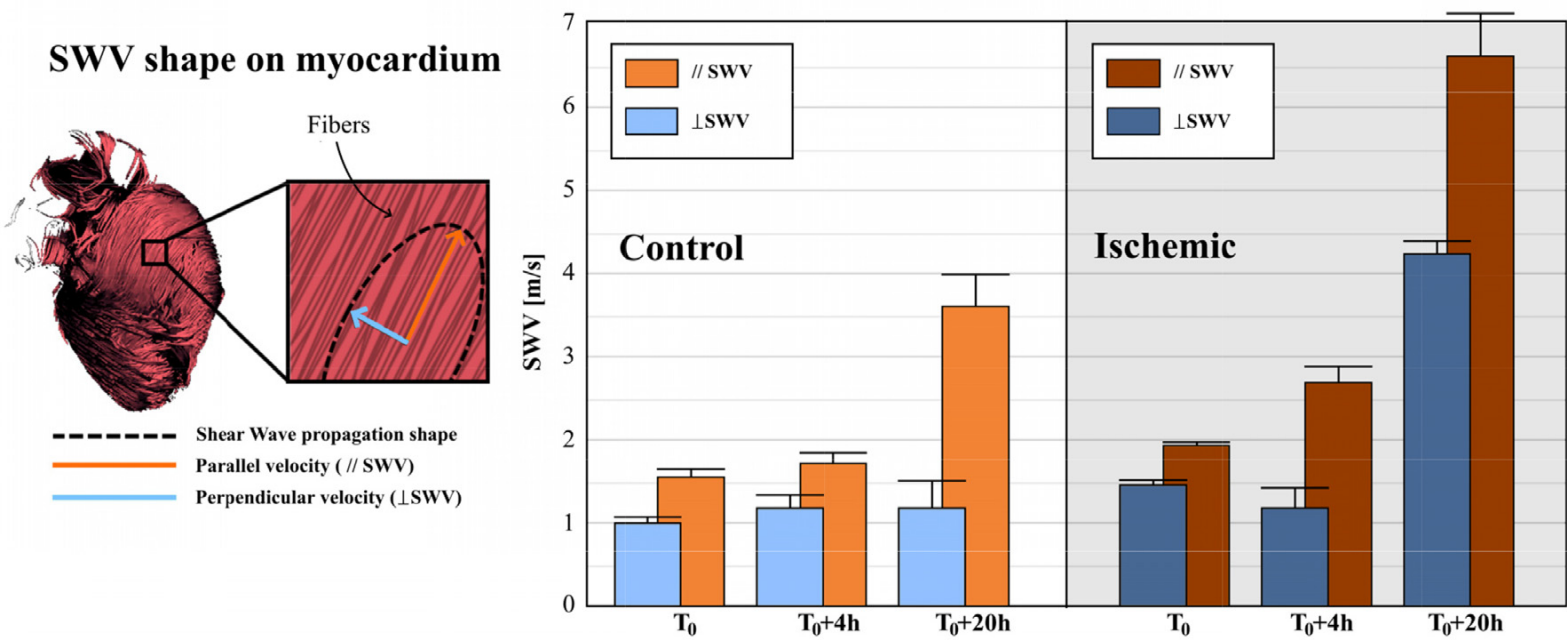
On the left, representation of the SWV shape on myocardium, with the parallel and perpendicular SWV represented by the arrows. On the right, the evolution of the parallel and the perpendicular SWV along time on two control and two ischemic cardiac grafts.

Parallel and perpendicular SWV in two control and two ischemic hearts taken from the previous studied sets are presented on Figure 6. It should be noted that the values are consistent with the measurements reported in sections B and C using Aixplorer. Figure 6 shows that the perpendicular velocity remained unchanged in both control and ischemic groups until 4h whereas the parallel velocity significantly increased in the ischemic group but not in the control group. It suggests that the early stiffening observed for the ischemic group could be mainly due to the stiffening in the direction of fibers but not to the stiffening in the perpendicular direction (i.e the extracellular matrix). At 20h, on the ischemic model both parallel and perpendicular velocities significantly increased. Due to a small number of cases a significant testing could not be computed.

### Correlation of myocardial stiffness and post-revival cardiac function

Two hearts of the two groups were reperfused after 4h and 20h of preservation to evaluate the cardiac function using multifactorial scores, (Table 1). We correlated the global SWV measured at the same time

**Table 1:** Post reviving score of heart graft versus SWV values.

Model	Preservation time [h]	RPP [mmHg/min]	Myocardial hardening	Contractility rate [mmHg/s]	Relaxation rate [mmHg/s]	EDP[mmHg]	SWV [m/s]
**CONTROL**	4	14,445.86	0	1908.29	1281.29	16.29	1.69 +/−0.19
**CONTROL**	20	10,584.33	0.63	112.00	963.00	17.40	3.40 +/−0.75
**ISCHEMIC**	4	4866.00	1.56	1259.00	945.00	15.50	3.04 +/−0.69
**ISCHEMIC**	20	0.00	3.55	0.00	0.00	60.00	4.77 +/−1.22

The SWV values correlate well with all the cardiac function parameters as shown in Table 2. A high correlation coefficient was found for the contractility rate who estimates the contraction efficiency (r^2^=0.88), the myocardial hardening from palpation performed by two operators (r^2^=0.81) and the relaxation rate (r^2^=0.81). The other parameters also showed a correlation with SWV values, the Rate Pressure Product (RPP), the product between heart rate and systolic blood pressure used to determinate the graft workload and the End Diastolic Pressure (EDP) have R squared scored of 0.77 and 0.67, respectively.

**Table 2:** Correlation between the reviving indexes and SWV.

		RPP(LVDP x HR)	Myocardial hardening	Contractility rate	Relaxation rate	EDP
**Pearson r**	SWV	0.88	0.90	−0.90	−0.94	0.82
**R squared**	SWV	0.77	0.81	0.81	0.88	0.67

## Discussion

In this study, we assessed the graft stiffness using elastography technique to predict its viability on preclinical situation close to human setting. A dedicated setup for ultrasound imaging and SWE was developed to image whole explanted hearts and assess quantitatively the myocardial stiffness over time.

Pig heart (*N*=14) were assessed in two groups: a control group harvested in normal conditions and an ischemic group with 30 min of *in situ* warm ischemia before harvesting to mimic a DCD situation. Each model was studied during 20h to evaluate the stiffness alterations due to ischemia and to preservation time. The study was performed at a preservation temperature of 4°C. Finally, we found a strong correlation of the SWV values with cardiac function parameters measured when the hearts were revived. The anatomy and physiology of the porcine heart is close to the human heart. The surgical procedures used were very similar to those used in the clinical setting.

Myocardial stiffness is a biomarker which reflects the tissue architecture and its content at macroscopic and microscopic levels. However, the precise mechanism of graft deterioration and stiffness alterations during cold ischemic time is not yet elucidated. In deep hypothermia (4°C), heart is non-beating and cardiac metabolism is reduced by more than 95%. The oxygen demand is therefore identically reduced. Essentially an ion imbalance occurs between the intra and extracellular environments, with:

(1) an entry of Na+ into the cells (following the reduced activity of cold sensitive Na/K-ATPases). This entry of Na+ is accompanied by a concomitant entry of water, which induces edema and therefore hardening of the tissues.
(2) Ca++ entry due to the dysfunction of calcium regulatory pathways. This calcium invasion induces a progressive contracture of the cardiac myofibrils causing an increase in the rigidity of the myocardium. This phenomenon can be activated by the decrease of energy stores (ATP, PC), that gradually keep the myofibrils in a contraction state.

These phenomena are intricated and occur globally throughout the myocardium, perhaps more marked in denser cellular areas such as the septum and the left ventricle. We can therefore expect to observe a progressive hardening of the graft with the time of ischemia, correlated with the suffering of the organ. This is precisely what clinicians try to assess qualitatively by manual palpation and what we measure quantitatively by elastography.^30^, ^31^

Previous studies have shown that myocardial stiffness measured *in vivo* by SWV was significantly altered by age and pathologies such as hypertrophic cardiomyopathy,^29^ amyloidosis^25^^,^^36^ and after heart transplant. We show that SWV is a reliable tissue marker for monitoring the organ preservation as a function of time. In control hearts, myocardial stiffness remained unchanged for 4h of preservation but increased significantly between 4 and 20h, which is consistent with the recommendations for preservation duration (which is usually less than 4h on average in clinical practice). In contrast, 30-min ischemic hearts were found to be initially stiffer and to stiffen prematurely at 4h suggesting an early alteration of the myocardium.

Because, the myocardium is composed of fibers locally oriented in a principal direction, the myocardial elastic properties are anisotropic.^37^^,^^38^ Analysis of the elastic anisotropy was performed using a dedicated prototype for cardiac elastography and the results showed that stiffness alterations at 4h for the ischemic group were observed mainly in the direction of fibers, whereas the perpendicular SWV remained unchanged. It suggests that early stiffening of ischemic hearts (< 4h) was related to the myocardial contracture. In contrast, the strong stiffness increase observed at 20h in both groups was linked to the increase of parallel and perpendicular SWV which suggests that all components including the myocytes and the extracellular matrix have been altered. The variation of visco-elastic properties could also be interesting to study. It will require the use of dedicated SWE acquisitions.

SWV was found to correlate strongly with the parameters of the cardiac function after reviving the hearts suggesting that SWV could be used as a marker of myocardial viability and a predictive marker of the cardiac function. The normal SWV was around 1.7 m/s, and a myocardial stiffness of about 3.0 m/s and higher was associated with a significant decrease of the cardiac function. Above 4.5m/s the hearts were definitively damaged and could not be restarted.

Various strategies have been proposed to expand the donor pool^3^^,^^4^ but many potential donors do not meet the criteria for transplantation. One of the most promising avenues to increase the donor pool is that of using grafts from patients who died following a planned cessation of care. These patients (known as Maastricht-3 or M-III in the European Union) therefore die of cardiopulmonary arrest in a hospital setting. On some M-III patients, it is possible to remove organs for transplantation, at the express condition that a cardiorespiratory downtime of approximately 30 min is observed. These potential grafts have therefore an initial ischemic pain (global warm ischemia) of at least 30 min before harvesting. Increasing organ availability with donation after circulatory death (DCD) could be a promising option to overcome the shortage of cardiac grafts.^5^, ^6^, ^7^ However, potential organ damages induced by ischemia have raised concerns for the graft. We show here that the SWE offers a rapid, non-invasive and quantitative tissue marker of heart graft quality. This could become a powerful tool to optimize preservation strategies and also for graft viability estimation, especially those from DCD. Possibility of real time non-invasive measurements associated with easy implementation, strongly suggests that this technique may be considered for a future using during the transplantation process. SWE has the potential to provide a tissue biomarker of the heart viability and could offer a new accessible, portable and predictive tool before heart transplant. The evaluation of myocardial stiffness could also offer a new marker of the evaluation of cardiac function after transplantation. This will allow a new perception of the graft transplantation process and its impact on the patient health.

One limitation of this study is the small sample size, to determine precise cutoff values, further validations will be required on animal and human hearts including measurements at other preservation times in particular before the 4h of preservation and after the reperfusion of the organ. One should note that shear wave velocity and myocardial stiffness are slightly dependent on preservation temperature (see supplementary data). The acquisition of the entire heart required 20 min of scanning, this duration could be significantly reduced by an optimization of the scan parameters (Number of slices, spatial step between the slices) or by reducing the scan to 2 or 3 box facets.

Before considering SWV as a marker of graft viability for clinical transplantation, the study needs to be translated to human grafts to validate the use of SWV as biomarker on real human hearts. SWE measurement needs to become widely available in dedicated devices for transplantation applications. The development of algorithms and precise SWV cut off values will be also required to propose clear guidelines on the use of SWV. Quantification of myocardial properties could be crucial in order to optimize preservation strategies, harvesting technique and finally to expand the number of grafts available for cardiac transplantation. The methodology developed in this work is relatively affordable compared to MRI systems, and can provide noninvasive whole heart scans in a very short time (few minutes), compatible with urgency encountered on clinical practice. Clinical ultrasound devices with shear wave elastography modality are widely available for several manufacturers and therefore, this approach, could be directly translated to the clinics.

## Contributors

O.P., C.P., R.F. and M.P. did the concept and study design. L.A., J.L., M.L.G. and R.F. acquired data. O.P., C.P. and M.P. performed data processing. Writing, review, and/or revision of the manuscript: O.P., C.P., R.F. and M.P. All authors reviewed and commented on the manuscript, and approved its final submission. Underlying data have been verified by C.P and M.P.

## Data sharing statement

Data that support the findings of this study will be available from the corresponding author of the study.

## Declaration of interests

All authors have no conflicts of interest to disclose.

## Appendix Supplementary materials

Image, application 1
