# Supplementary figures and images for: Quantitative stiffness assessment of cardiac grafts using ultrasound in a porcine model: A tissue biomarker for heart transplantation
Source: eBioMedicine. 2022 Aug 3;83:104201. doi: 10.1016/j.ebiom.2022.104201 (PMC9358428; doi:10.1016/j.ebiom.2022.104201)

## Relation between temperature and shear velocity

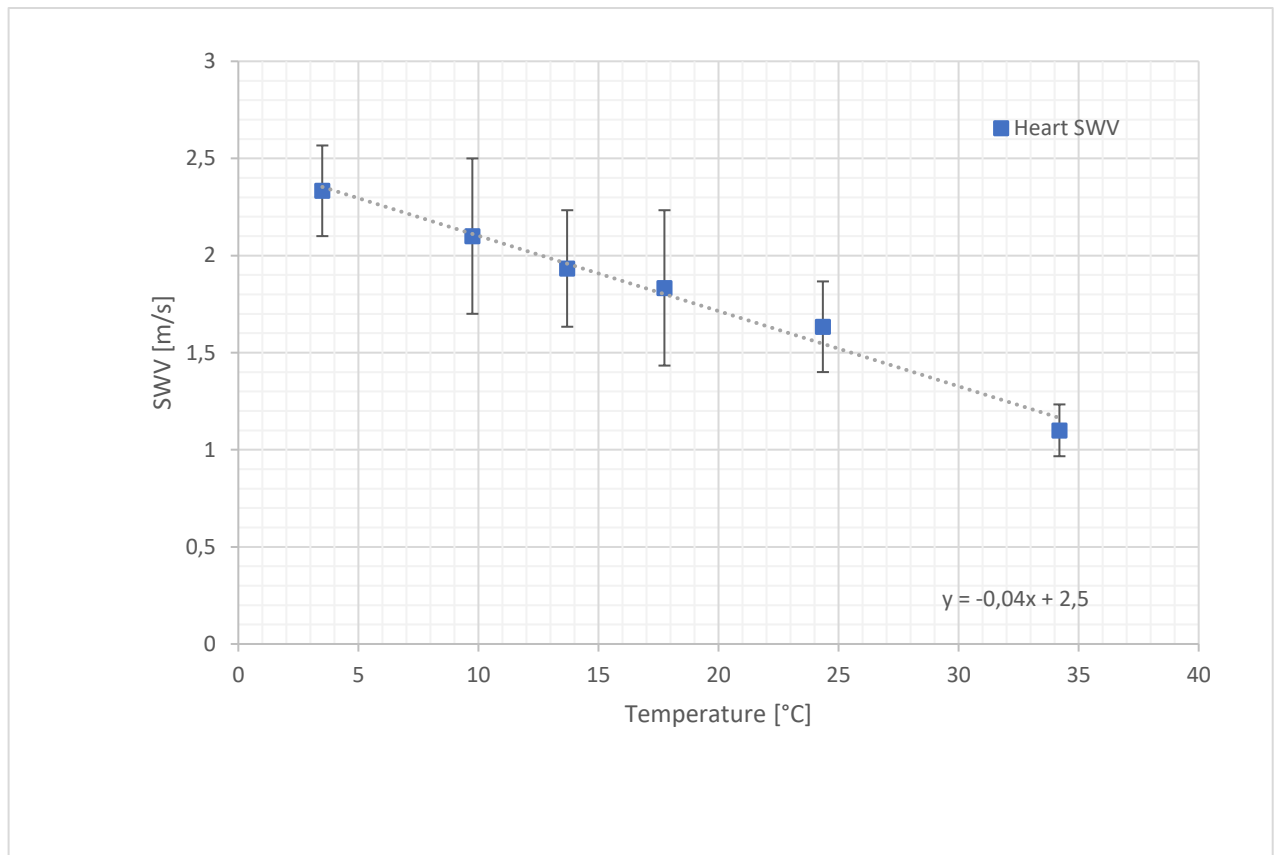

Supplement: Supplementary file 1 [file mmc1.pdf]
